# Investigation of structural parameters influencing vibration characteristics of heavy-duty truck diesel tanks

**DOI:** 10.1371/journal.pone.0304712

**Published:** 2024-06-26

**Authors:** Xiaodong Hu, Yonglu Pi, Kedong Wang, Xue Zhang, Qiang Zhang, Mingfei Mu

**Affiliations:** 1 College of Mechanical and Electronic Engineering, Shandong University of Science and Technology, Qingdao, China; 2 School of Intelligent Manufacturing, Qingdao Huanghai University, Qingdao, China; NED University of Engineering and Technology, PAKISTAN

## Abstract

The working conditions of heavy-duty trucks are very complicated as the diesel shaking and resonance problems, which causes weld tears, separators to fall off, and other failures occur. Through experiments and finite element simulation, the natural frequency and vibration mode of a given 400 L diesel tank were calculated to study the influences of structural parameters such as the fill ratio (0.1–0.9), the number of baffle plates (0, 1, 2), the spacing of the plates (240 mm, 400 mm, 560 mm) and the aperture (38 mm, 78 mm, 118 mm) on the modal parameters with the wet mode method. The results of the hammering mode test and the simulation modal analysis agree well with the maximum error is 4.8%; the natural frequency of the diesel tank will increase with fill ratio decrease; the increase of the baffle plate number (0, 1, 2) can effectively increase the first-order natural frequency of the diesel tank, but the change of the natural frequency is not obvious on the higher order; the higher plates spacing has a smaller natural frequency; increasing the aperture will highly increase the natural frequency, 188 mm has better vibration safety.

## 1. Introduction

The driving process of heavy duty trucks is accompanied by various complex conditions, such as acceleration, deceleration, and turning, which give rise to the internal problems of the diesel tank, including fluid sloshing and intricate vibration. These problems can lead to faults such as weld seam tearing and baffle plate detachment, and even fragments of aluminum-magnesium alloy materials can follow the oil into the engine, posing a threat to the safety of the engine and the entire vehicle [1, 2].

With the aim of influencing the structure of the tank and the movement of the internal fluid, the researchers have conducted a lot of research by experiments and simulations [3]. Jhung et al. investigated the modal characteristics of a square water tank using the finite element method, evaluating the influence of fluid and boundary conditions on the modal properties to ensure its structural integrity under dynamic loads [4]. Shen et al. studied the vibration free-coupled of partially filled baffle plate tanks with elastic thin plates, considering different types of fluid-coupled elastic thin plates and further revealing fluid sloshing modes inside the tank [5]. Hu et al. explored the influence of stiffener design, number, and position on the modal frequency of cylindrical shell. It is found that the number of stiffeners has a converging effect on the modal frequency, the height of the stiffener section is more sensitive to the modal frequency of the cylindrical shells than the width of stiffener section [6]. Liu et al. established a numerical model for simulating fluid sloshing using the multiphase flow and dynamic mesh method and studied the impact of different gravitational accelerations on the dynamic characteristics of fluid sloshing [7]. Gao et al. used the acoustic structural coupling algorithm to analyze the wet modalities of the structure under different internal and external pressures, and changes in its natural frequency and vibration mode under different internal and external pressures [8]. Zheng et al. proposed a topology optimization method to reduce the fuel sloshing effect, a mathematical model of hybrid fluid-solid coupling was built based on solid and fluid SPH particles, which optimized the hole layouts on the tank rib to reduce fuel sloshing time, increase stiffness and guarantee lightweight requirements [9]. Combined with fluid-structure interaction theory, Wang et al. compared the axial flow changes of the impeller modes in water and air under pre-stress conditions, the water has a significant reduction in the natural frequency of the impellers, while pre-stress has no obvious effect on the mode [10]. Strelnikova et al. analyzed the vibration response of rigid cylindrical liquid-filled tanks under vertical and horizontal loads, considering the effects of horizontal and vertical baffles and elastic effects, and studied the tank response under free vibration, forced vibration, and parametric vibration [11].

The above research involves the results of finite element analysis [12], computational fluid dynamics [13, 14], fluid-structure interaction [15, 16], and vibration [17, 18], etc., which provides a new method for the design of tanks like diesel tanks, reduces development costs, and improves product quality [3]. However, there are still deficiencies in the research on the sloshing problem of diesel truck tanks, and the influence of fill ratio and baffle plate on the vibration characteristics of diesel tanks is not considered comprehensively.

In this article, the natural frequency of a 400 L diesel tank is calculated by combining experiment and simulation analysis. The influence of different fill ratio, number of baffle plates, baffle spacing, and baffle aperture on its vibration characteristics is studied, further refining the design methods for truck diesel tanks and providing a theoretical basis for the upgrade and modification of truck diesel tanks.

## 2 Modeling of diesel tank

**Fig 1:**
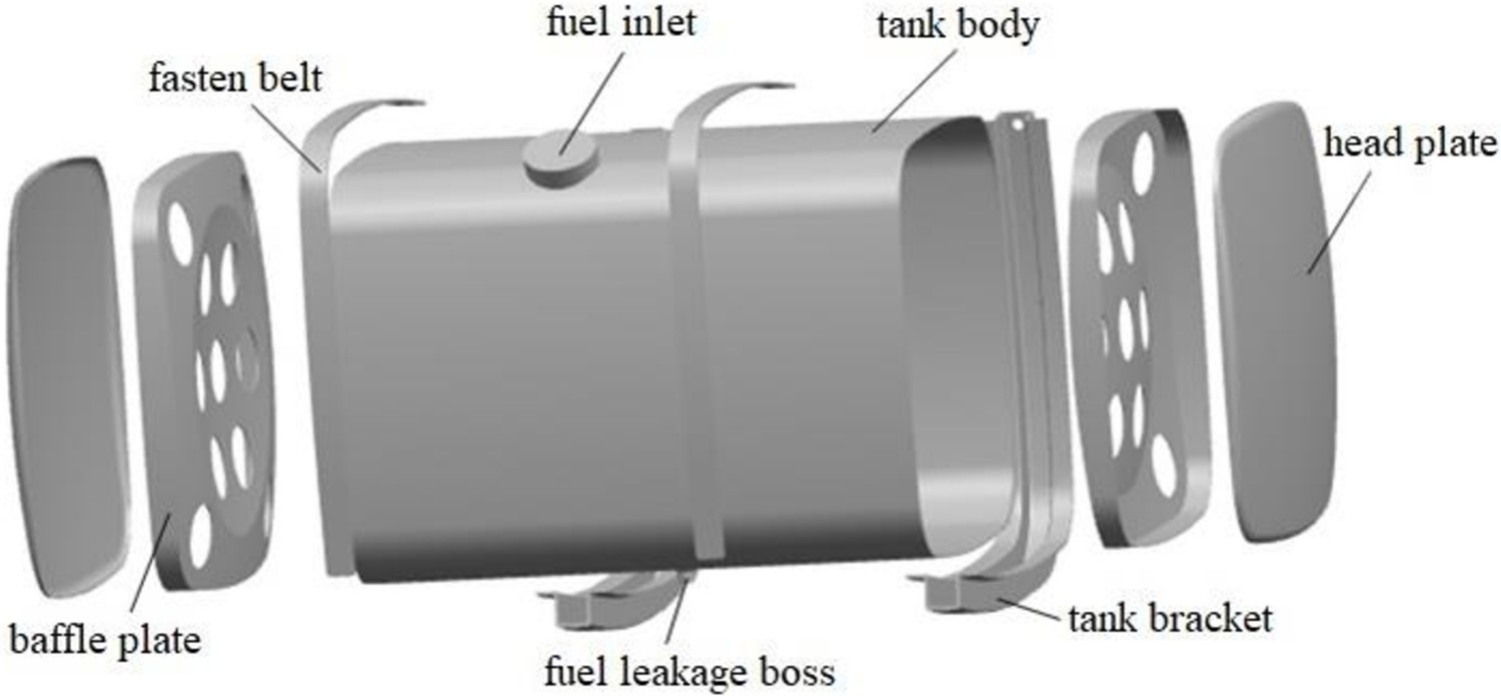
Structure diagram of the diesel tank.

The structure of the diesel tank of the truck mainly includes the tank body, head plate, baffle plate, tank bracket, fuel inlet, fuel leakage boss, etc. The tank body and baffle material is aluminum-magnesium alloy, and the bracket is made of automobile beam steel, as shown in Fig 1. The tank has a capacity of 400 L, with dimensions of 965 mm in length, 680 mm in width, 650 mm in height, and a thickness of 2.5 mm. The thickness of the baffle plate is 2 mm. The materials and mechanical properties are provided in Table 1.

**Table 1:** Material property.

Part	Material	Density (kg/m^3)^	Elastic modulus *E* (GPa)	Poisson ratio
Tank body	5052	2680	70	0.33
Baffle plate	3003	2730	69	0.33
Tank bracket	510L	7853	211	0.29

## 3 Modal test of the diesel tank

### 3.1 Experiment

**Fig 2:**
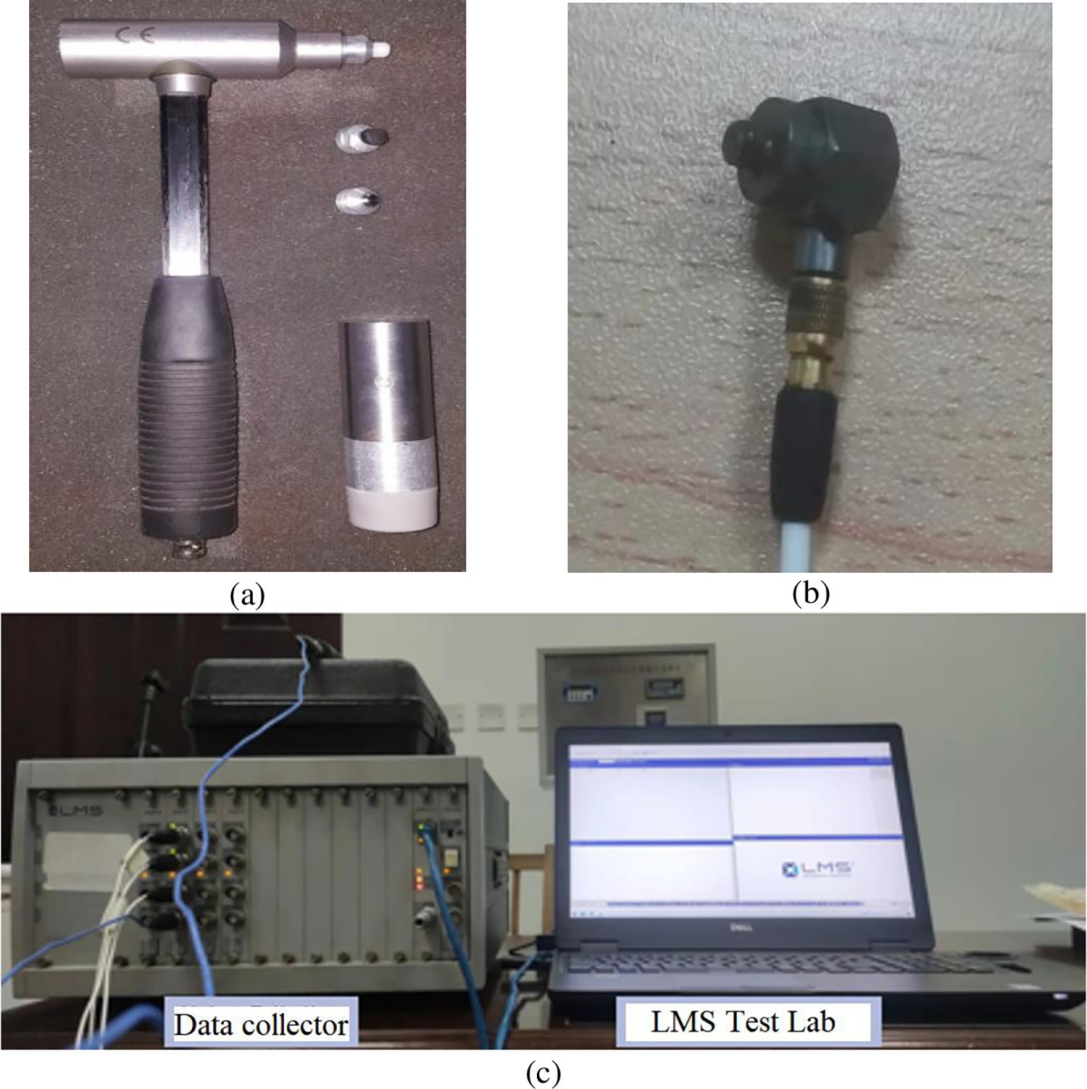
(a) Nylon hammer (b) acceleration sensor (c) LMS system.

**Fig 3:**
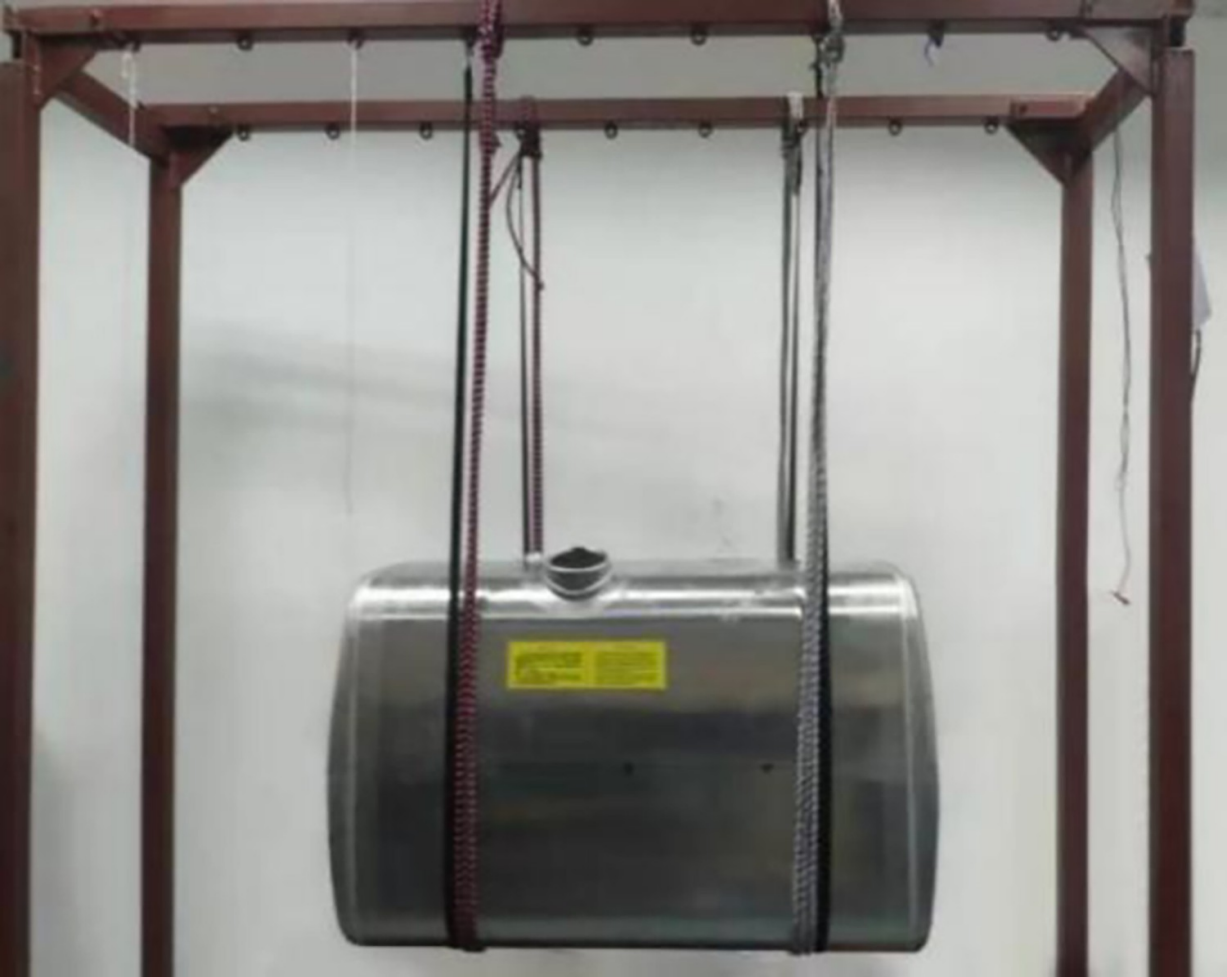
Free suspension of the diesel tank.

The vibration characteristics of the diesel tank were measured using a method of tapping multiple points and collecting single points of data. During the tapping process with a force hammer, excitation signals were simultaneously collected. Data acquisition was performed using the Impact Hammer Modal Testing module in the LMS Test. Lab software, as can be seen in Fig 2. The diesel tank structure was then modified using the Modal Analysis module. When measuring the vibration characteristics of the diesel tank in the free state, the elastic damping rope is used to suspend the tank to realize the free boundary conditions, and the accuracy of the vibration mode calculated by the finite element method is verified. The specific suspension setup is illustrated in Fig 3.

### 3.2 Vibration characteristics test results

**Fig 4:**
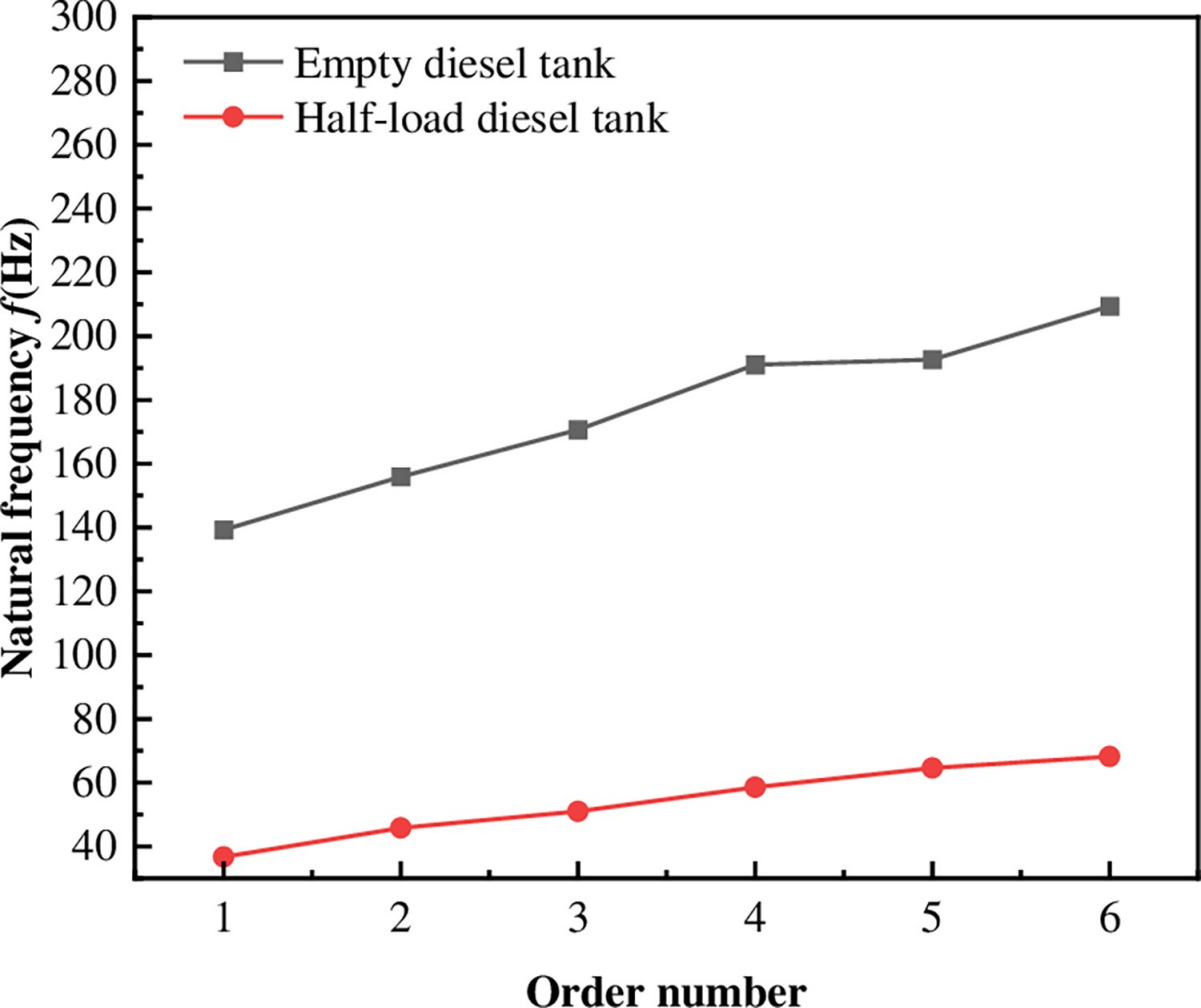
Modal test data of an unconstrained diesel tank.

Modal tests were conducted in diesel tanks without oil and half-filled. The collected data were processed and analyzed by Time MDOF. The frequency response functions (FRFs) were computed to determine the system’s first six natural frequencies, as depicted in Fig 4. The natural frequencies of the half-load diesel tank can be found to be much lower than those of the empty diesel tank.

## 4 Vibration characteristics simulation of the diesel tank

### 4.1 Grid independence

**Fig 5:**
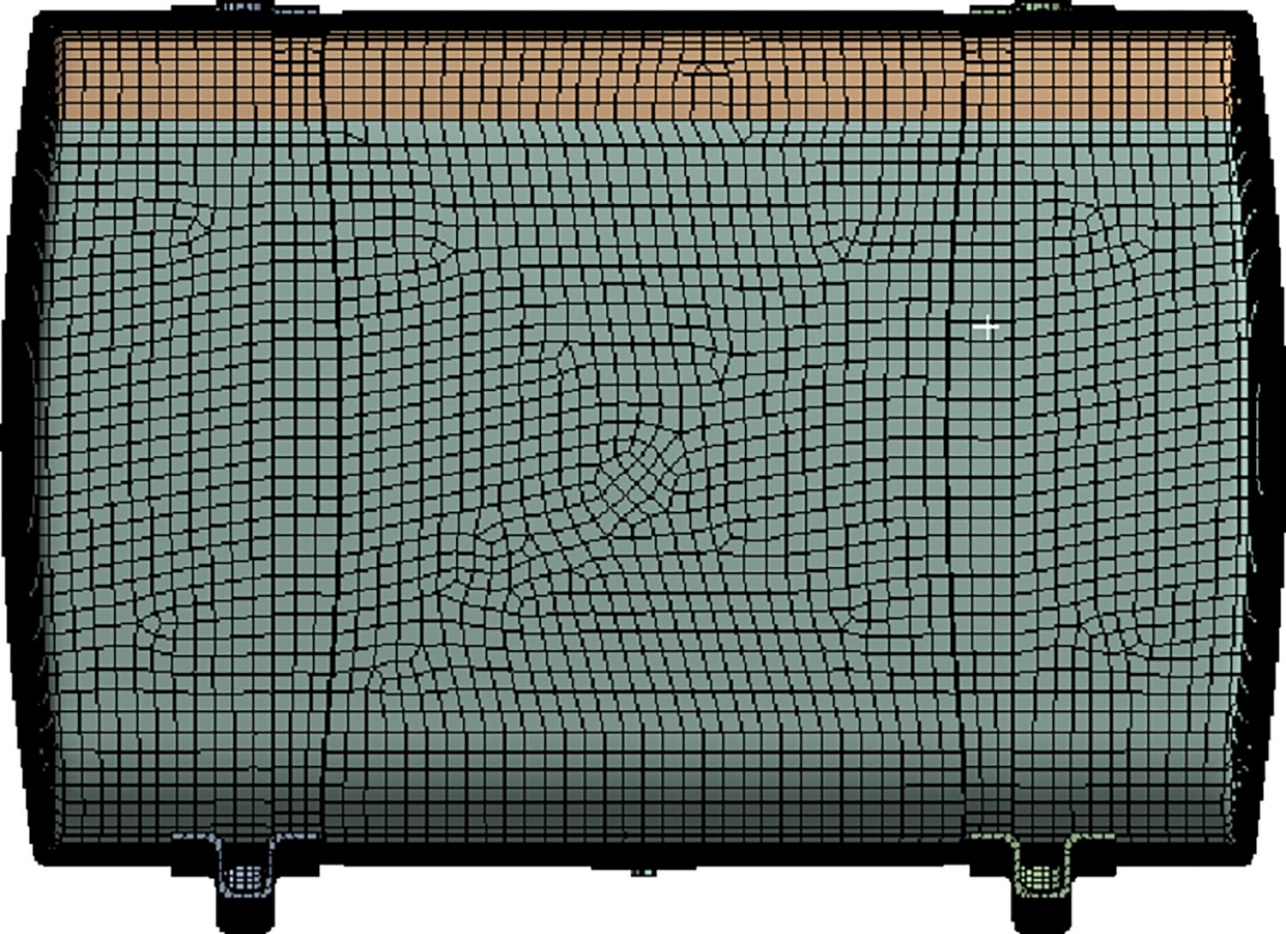
Grid division of diesel tank.

The diesel tank grid division is illustrated in Fig 5. Due to the complexity of the internal regions of the diesel tank, considering the influence of grid quality on subsequent computational convergence accuracy and result precision, local refinement was applied to the boss and baffle plates. Three different grid numbers were generated, with counts of 372241, 546610, and 796239, respectively. The results indicated that as the number of the grid increases, the pressure variation at the monitoring point is minimal, with a relative error within 2%. Therefore, the final selection of the grid number was determined to be 372241. The minimum grid size was set at 2 mm, the maximum grid growth rate was set at 1.2, and poly-Hexcore volumetric grid technology was used to achieve precise grid partitioning.

### 4.2 Theoretical model

The simulation calculation process involves the fluid dynamics control equations and fluid-structure interaction. Typically, fluid dynamics equations consist of the continuity equation, momentum equation, and energy equation [19]. This study does not involve temperature calculations, so the energy equation is not considered.

#### 4.2.1. Continuity equation

The continuity equation is also an equation of mass conservation. It expresses that within a fluid domain, the mass entering the domain per unit time is equal to the mass exiting the domain. This relationship is formulated as Eq (1).

$$\frac{\partial \rho }{\partial t}+\frac{\partial (\rho u_{x})}{\partial x}+\frac{\partial (\rho u_{y})}{\partial y}+\frac{\partial (\rho u_{z})}{\partial z}=0$$ (1)

Where, *u*_*x*_, *u*_*y*_ and *u*_*z*_ is the velocity component in the x, y, z directions, m/s; *t* is time, s; *ρ* is density, kg/m^3^.

If it is a steady flow, $\frac{\partial \rho }{\partial t}=0$; if it is incompressible fluid, *ρ* is constant. For these two cases, the continuity equation can be written as follows.


$$\frac{\partial u_{x}}{\partial x}+\frac{\partial u_{y}}{\partial y}+\frac{\partial u_{z}}{\partial z}=0$$ (2)

#### 4.2.2. Momentum equations

The momentum conservation equation is a fundamental law that must be followed when the fluid is in motion within a fluid-structure interaction system. The change rate of the fluid momentum in time is equal to the sum of the external forces acting on the fluid. This equation is also known as the fluid motion equation, or the Navier-Stokes equation, represented by Eq (3).

$$\{\begin{array}{c} \rho \frac{du}{dt}=\rho F_{bx}+\frac{\partial p_{xx}}{\partial x}+\frac{\partial p_{xy}}{\partial y}+\frac{\partial p_{zx}}{\partial z}\\ \rho \frac{du}{dt}=\rho F_{by}+\frac{\partial p_{xy}}{\partial x}+\frac{\partial p_{yy}}{\partial y}+\frac{\partial p_{zy}}{\partial z}\\ \rho \frac{du}{dt}=\rho F_{bz}+\frac{\partial p_{xz}}{\partial x}+\frac{\partial p_{yz}}{\partial y}+\frac{\partial p_{zy}}{\partial z} \end{array}$$ (3)

Where, *F*_*bx*_, *F*_*by*_ and *F*_*bz*_ is the mass force component in the x, y and z directions, N; *p*_*xx*_, *p*_*xy*_ and *p*_*xz*_ is the component of the stress tensor in a particle in the fluid.

#### 4.2.3. Fluid-structure interaction governing equation

To ensure the accuracy of the fluid-structure interaction, it is imperative to ensure its compliance with multiple conservation laws. Therefore, the fluid-structure interaction interface should satisfy the equality and conservation of a series of variables, such as displacement *d*, heat flux *q*, fluid and solid structural stresses *τ*, temperature *T*, etc. [20, 21]. These relationships can be expressed using the following formulas:

$$d_{f}=d_{s}$$ (4)

$$q_{f}=q_{s}$$ (5)

$$\tau_{f}\cdot n_{f}=\tau_{s}\cdot n_{s}$$ (6)

$$T_{f}=T_{s}$$ (7)

In the practical simulations, as the fluid domain and structural domain grids are partitioned separately and there is no one-to-one correspondence between the nodes of the grids, direct data exchange between them is not possible. Therefore, a specific mapping method must be employed to define the mapping operators at the fluid-structure interaction interface, as in Eq (8).

$$\left\{ \begin{array}{l} Y_{\mathrm{sf}}:\{f_{i}|i\in N_{f}\}\to \{{\hat{f}}_{i}|j\in N_{s}\}\\ Y_{\mathrm{fs}}:\{f_{j}|j\in N_{s}\}\to \{{\hat{s}}_{i}|i\in N_{f}\} \end{array}\right.$$ (8)

Where, *f*_*i*_ and *f*_*j*_ is the physical quantities on the fluid and solid nodes respectively; $\hat{f}$ and $\hat{s}$ is the interpolation of the physical quantities of the structure and fluid nodes on the fluid-structure interaction surface, respectively. $\hat{f}=M_{\mathrm{sf}}f$, $\hat{s}=M_{\mathrm{fs}}s$.

#### 4.3 Modal analysis under unconstrained boundary conditions

**Fig 6:**
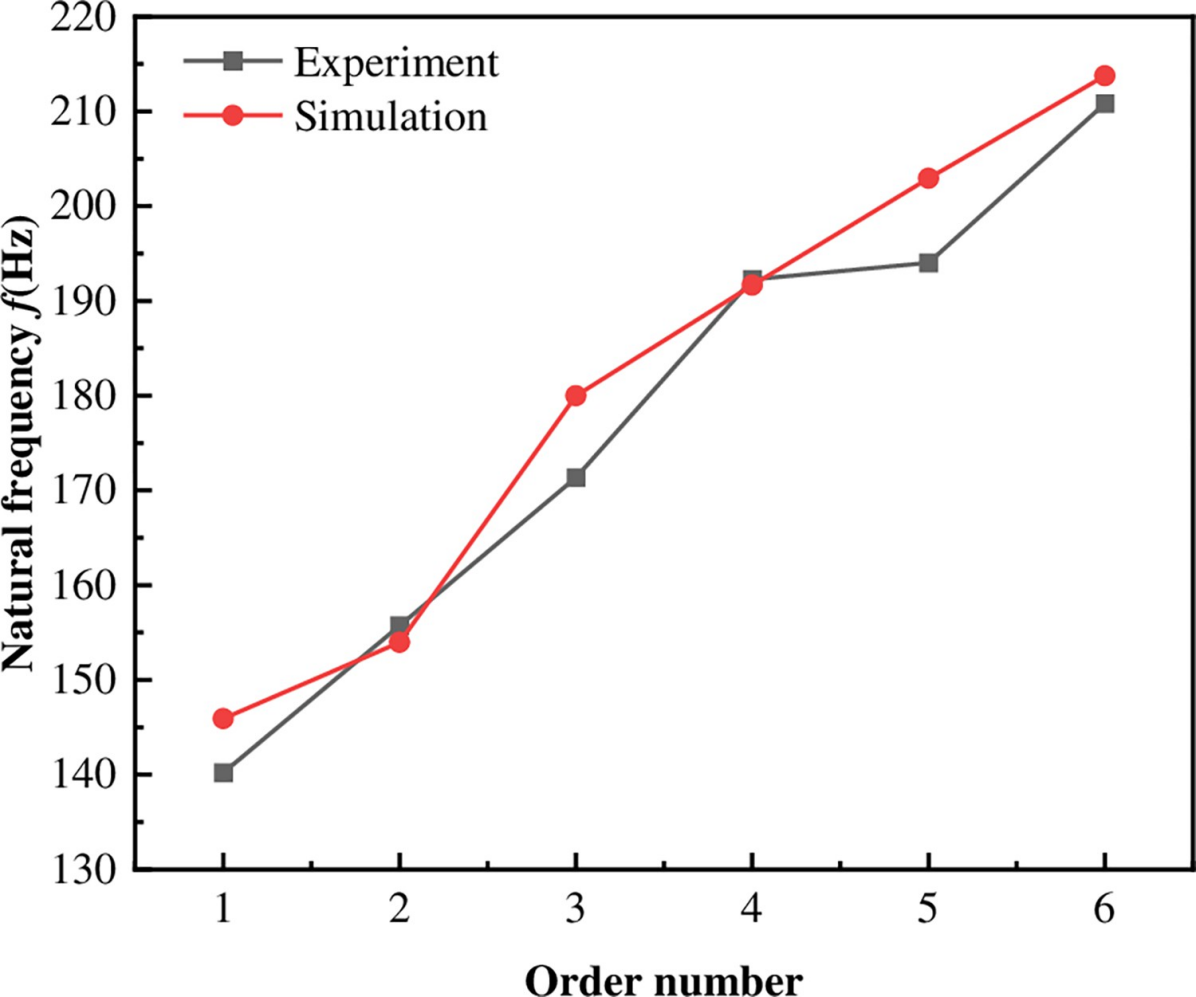
Comparison between unconstrained modal simulation and diesel tank test results.

Comparison of natural frequencies obtained from the structural simulation and experiment of the diesel tank under unconstrained boundary conditions, as shown in Fig 6. The errors between simulation and experiment from the first to sixth are within 4.8%. The results of the simulated modes are in good agreement with those obtained from the experiment modes, which validates the feasibility of the simulation.

## 5 The influence of the fill ratio on the modal of the diesel tank

In the practical operation of diesel tanks, the internal fluid affects the natural frequencies, exhibiting typical wet mode conditions. To investigate the vibration characteristics of the tank under different oil volumes, a wet modal analysis was performed on diesel tanks with fill ratios of 0.1, 0.3, 0.5, 0.7, and 0.9, the analysis considered the additional mass of diesel and the contribution of flow field stiffness [22]. The influence of oil fill levels was studied by analyzing the natural and structural frequencies and modes. Simulation was conducted using the Modal Acoustics module in the ANSYS, with fluid materials modeled as air (1.225 kg/m^3^) and diesel (730 kg/m^3^), and the acoustic pressure of the free liquid surface was defined as 0. According to GB18296-2019 "Safety property requirements and test methods for automobile fuel tank and its installation" [23], the durability test of diesel tank vibration adopts a vibration frequency of 30 Hz, which is generally taken as the dangerous resonance frequency from 0.75 to 1.25 times, so it is determined that 22.5 Hz-37.5 Hz is the dangerous resonance frequency range.

**Fig 7:**
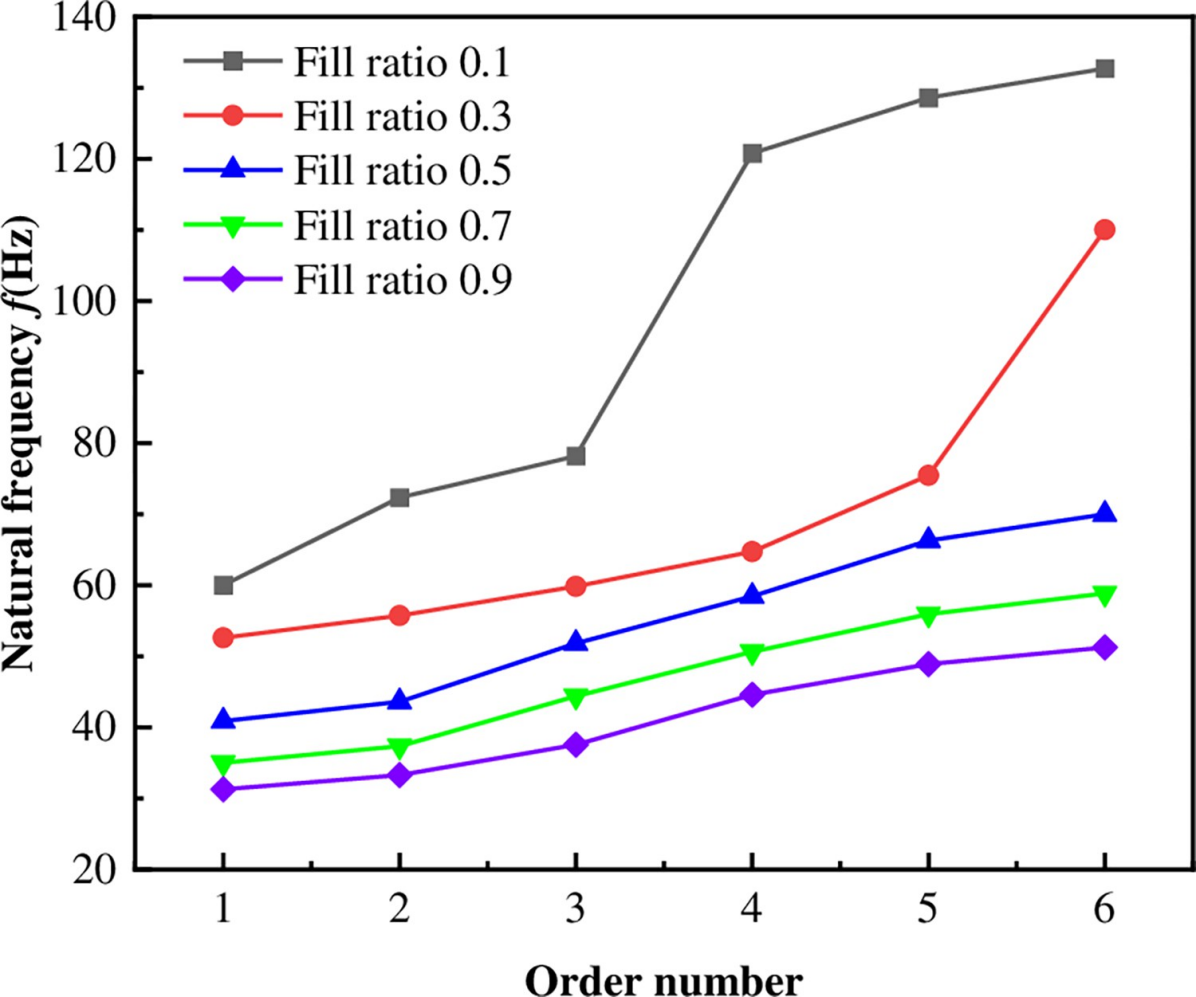
Natural frequency of diesel tank with different fill ratio.

The natural frequency curves under different fill ratios were as shown in Fig 7. Given that the fill ratio in the tank typically remains between 0.1 and 0.9 during most vehicle runs, the natural frequencies of the diesel tank with a fill ratio of 0.1 to 0.5 are not within the resonance frequency range. However, the first-order natural frequency with the fill ratio 0.7 is within the range, the first-order and second-order natural frequencies with the fill ratio 0.9 are 31.131 Hz and 33.326 Hz, respectively, which are within the range.

With increasing fill ratios, the natural frequency of the structure of the diesel tank continuously decreases, so the 0.9 fill ratio diesel tank was selected for wet modal analysis. Additionally, if the natural frequency of the diesel tank exceeds the dangerous resonance frequency range under the condition of a 0.9 fill ratio, as the truck drives and the oil decreases, the natural frequency will gradually increase. This ensures that the natural frequency of the tank remains within the safe range, avoiding resonance damage.

## 6 The influence of the baffle plate on the modal of diesel tank

The natural frequency of the diesel tank is influenced by the structure of the tank. To better understand the vibration characteristics of the diesel tanks under different structures, the baffle plate number, baffle spacing, and aperture are taken as the structure parameters to carry out the study.

### 6.1 The influence of the baffle plate number on the modal of diesel tank

**Fig 8:**
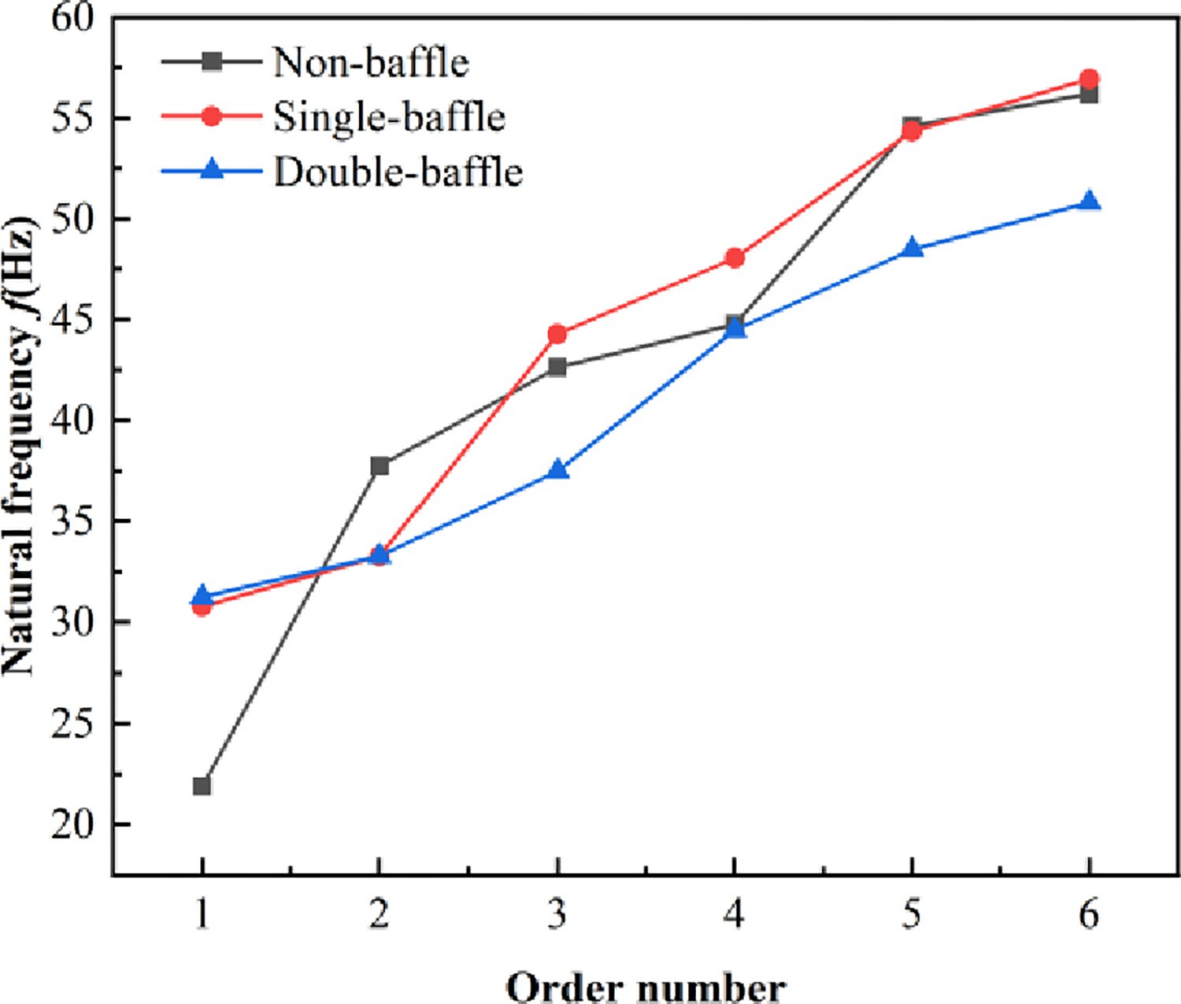
Natural frequency of diesel tank with different number of baffle plate.

Three tank structural configurations were considered: no baffle plate, a single baffle plate, and double baffle plates. A 0.9 fill ratio was established, the double baffle insert spacing was 400 mm, the aperture was 78 mm and other conditions were kept constant. The influence of the number of baffle plates on the vibration characteristics of the diesel tank was studied, as shown in Fig 8 and Table 2.

**Table 2:** Natural frequencies of first-six orders of diesel tanks with different numbers of baffles.

Order number	Non-baffle		Single-baffle		Double-baffle	
	Natural frequency *f* (Hz)	Maximum position of vibration amplitude	Natural frequency *f* (Hz)	Maximum position of vibration amplitude	Natural frequency *f* (Hz)	Maximum position of vibration amplitude
1	21.982	Tank body	31.019	Baffle plate	31.131	Baffle plate
2	37.785	Tank body	33.329	Baffle plate	33.326	Baffle plate
3	42.769	Tank body	44.309	Baffle plate	37.601	Tank body
4	44.883	Tank body	48.139	Baffle plate	44.72	Baffle plate
5	54.642	Tank body	54.431	Tank body	48.669	Baffle plate
6	56.504	Fuel leakage boss	57.04	Baffle plate	51.081	Baffle plate

It can be observed that as the baffle plate number increases from none to one, the increase of the first-order frequency is quite pronounced, from 21.982 Hz to 31.019 Hz. As the order increases, the differences between its natural frequencies are not as large as those of the first order. Although the first and second-order natural frequencies of the single baffle and double baffle diesel tanks are close, the changes in the higher order natural frequencies are more noticeable, and the natural frequency of the single baffle diesel tank is higher than that of the double baffle diesel tank.

**Fig 9:**
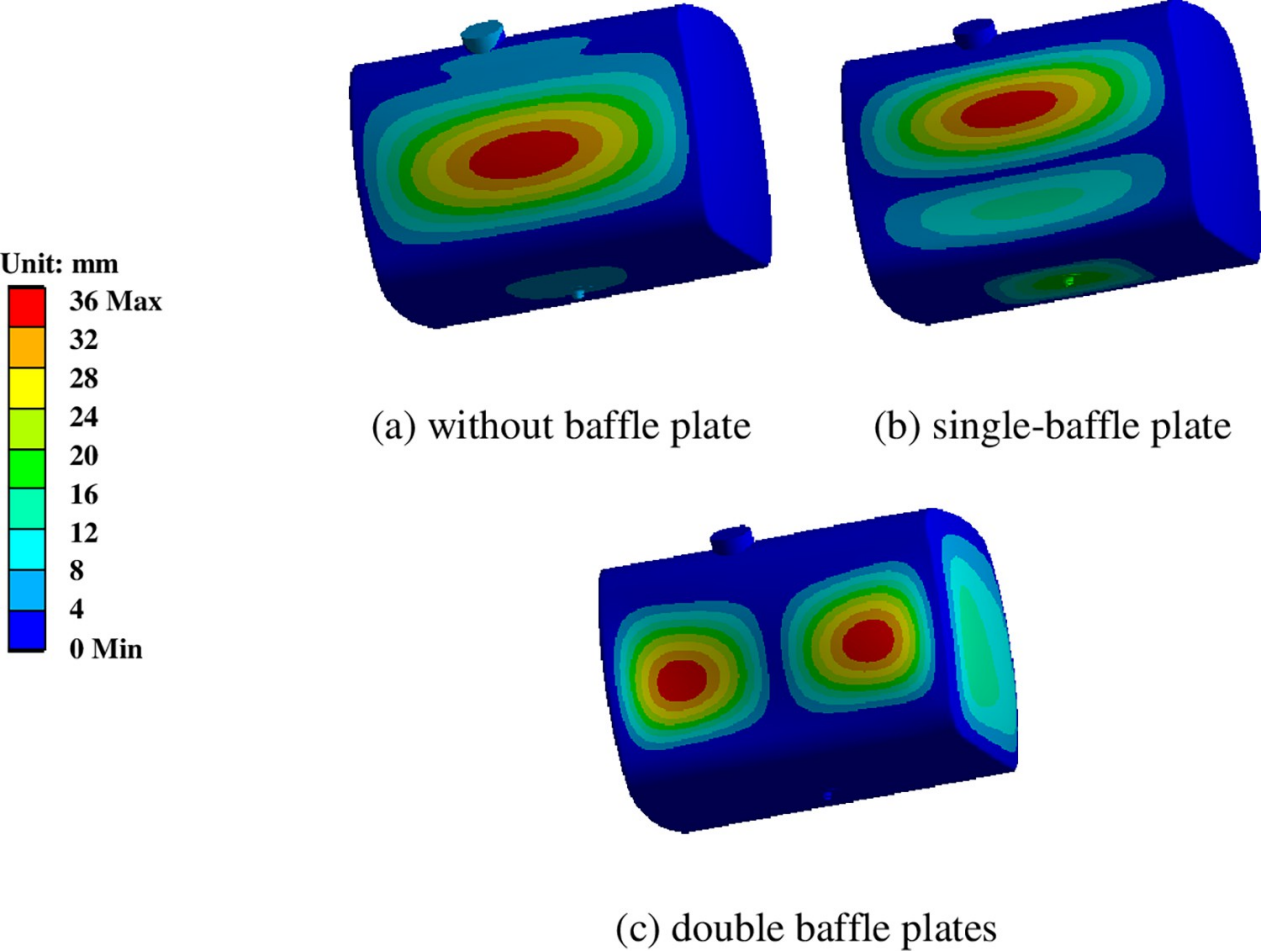
The fifth-order vibration mode of the diesel tank with different baffle plate number.

**Fig 10:**
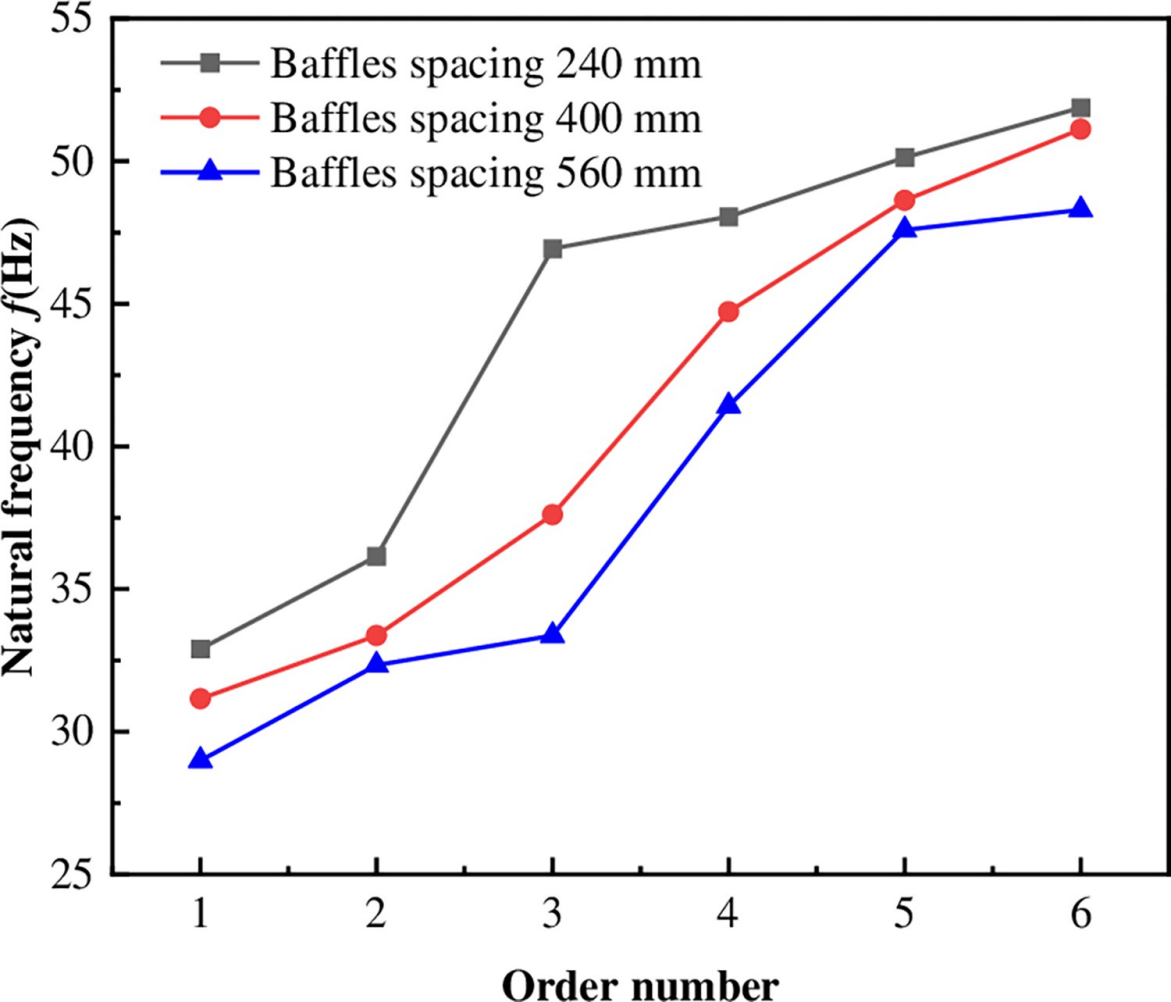
Natural frequency of the diesel tank with different baffle spacing.

**Fig 11:**
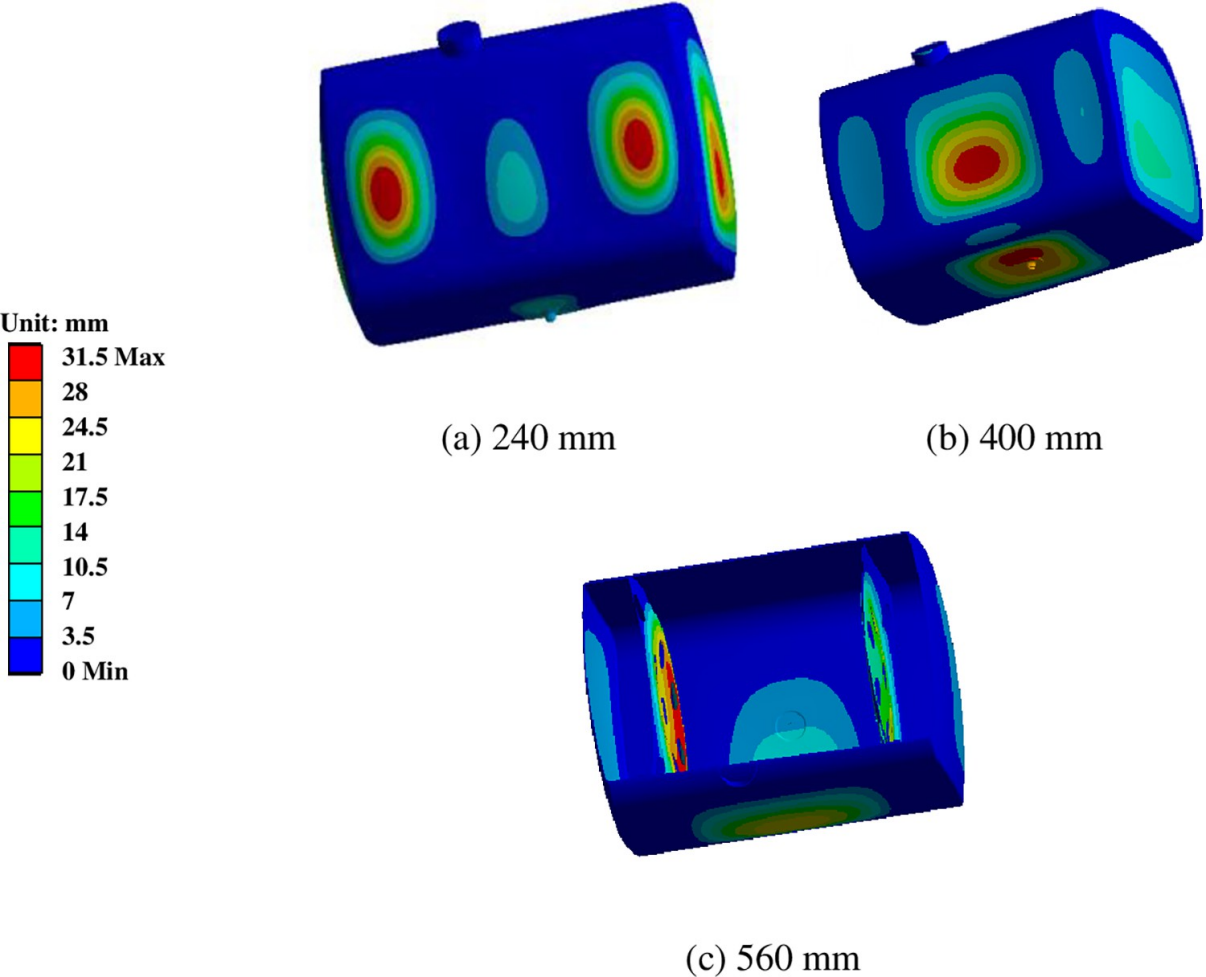
The third-order vibration mode of the diesel tank with different baffle spacing.

The first six vibration modes of the diesel tank with three baffle configurations are shown in Figs 9, 10 and 11. The deformation of the diesel tank without baffle is mainly concentrated on the middle side, there is no baffle support, with the order increasing, the vibration modes become slightly more complex, with the sixth-order vibration mode showing deformation primarily at the fuel leakage boss. The main deformation of the single- and double-baffle diesel tanks was on the baffle plates.

It can be observed in Fig 9, that the diesel tank without baffle relies mainly on the body and head plate as its primary structures, resulting in greater flexibility, and the maximum amplitudes are predominantly located on the tank body. However, for diesel tanks, a large sloshing impact occurs during vehicle operation; basically the impact is buffered by designing a baffle plate, so the baffle-free diesel tank is rarely used in practice. The diesel tank with single-baffle plate, characterized by an augmented baffle plate structure, predominantly experiences vibrational deformation concentrated within the vicinity of the baffle plate, with secondary manifestations observed along the periphery of the diesel tank. Conversely, in the case of the double baffle plates, vibrational deformation is principally governed by the baffle plate structure.

### 6.2 The influence of baffle spacing on the modal of diesel tank

When other conditions were kept constant for the diesel tank, with baffle spacing as a variable, modal simulations were conducted on diesel tanks with three different baffle spacings. The impact of baffle spacing on vibration characteristics was studied by analyzing the natural frequencies and vibration modes, as depicted in Fig 10 and Table 3. It was observed that as the baffle spacing increased, the natural frequencies of the diesel tank structure decreased, with a more noticeable reduction in the third order natural frequency, while the reduction in the natural frequencies of other orders was relatively small.

**Table 3:** The first six-order natural frequencies of diesel tanks with different baffle spacing.

Order number	240 mm		400 mm		560 mm	
	Natural frequency *f* (Hz)	Maximum position of vibration amplitude	Natural frequency *f* (Hz)	Maximum position of vibration amplitude	Natural frequency *f* (Hz)	Maximum position of vibration amplitude
1	32.981	Baffle plate	31.131	Baffle plate	29.018	Tank body
2	36.132	Baffle plate	33.326	Baffle plate	32.371	Baffle plate
3	46.941	Tank body	37.601	Tank body	33.32	Baffle plate
4	48.044	Baffle plate	44.72	Baffle plate	41.517	Tank body
5	50.047	Baffle plate	48.669	Baffle plate	47.543	Baffle plate
6	51.809	Baffle plate	51.081	Baffle plate	48.269	Baffle plate

The first six-order vibration mode of the diesel tank with baffle spacing 240mm and 560mm are illustrated in Fig 11. When comparing the vibration modes for the three baffle spacings, it can be observed that with an increase in baffle spacing, the locations of maximum deformation in the vibration modes also change. In the case of the diesel tank with a spacing of 560mm, the positions of maximum deformation for the first and fourth modes are no longer at the baffle plate but at the side of the tank body. Additionally, the increase in baffle spacing also shifts the maximum deformation location to the fuel leakage boss, making it a potentially hazardous location.

### 6.3 The influence of the baffle aperture on the modal of the diesel tank

Keeping the rest of the diesel tank structure unchanged, three different apertures (38mm, 78mm and 118mm) were designed for the double baffle plates with a spacing of 400mm and a fill ratio of 0.9.

**Fig 12:**
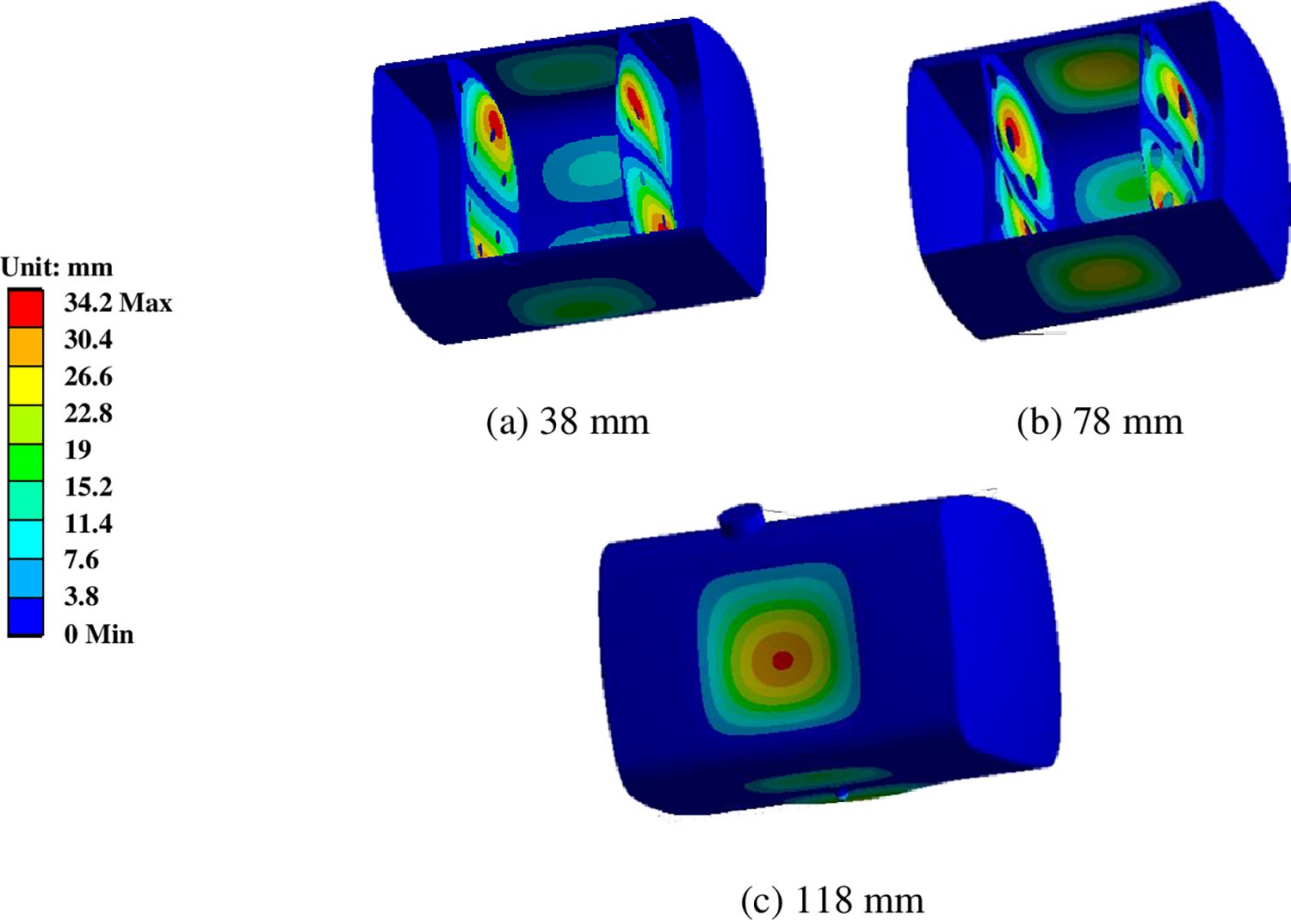
The fourth-order vibration mode of the diesel tank with different baffle aperture.

Fig 12 represented the first six-order vibration mode of the diesel tank with baffle apertures of 38 mm and 118 mm, and Table 4 presented the first six-order natural frequencies and the locations of maximum amplitude with apertures of 38 mm, 78 mm and 118 mm.

**Table 4:** First six-order natural frequencies of the diesel tank with different baffle apertures.

Order number	38mm		78mm		118mm	
	Natural frequency *f* (Hz)	Maximum position of vibration amplitude	Natural frequency *f* (Hz)	Maximum position of vibration amplitude	Natural frequency *f* (Hz)	Maximum position of vibration amplitude
1	21.886	Baffle plate	31.131	Baffle plate	36.576	Fuel leakage boss
2	24.551	Baffle plate	33.326	Baffle plate	44.551	Baffle plate
3	36.958	Tank body	37.601	Tank body	44.723	Baffle plate
4	39.232	Baffle plate	44.72	Baffle plate	48.176	Tank body
5	42.113	Baffle plate	48.669	Baffle plate	53.367	Baffle plate
6	47.378	Baffle plate	51.081	Baffle plate	56.509	Baffle plate

With an increase in the baffle aperture, the natural frequencies increase. For the diesel tank with a baffle aperture of 38mm, the first-order natural frequency is 21.886Hz, which is not within the resonance frequency due to its small size. However, the second-order and third-order frequencies are 24.551Hz and 36.958Hz, respectively, still fall within the range of the resonance frequency. The tank with an aperture of 78 mm still has the possibility of resonance at the first and second low-order vibrations, and with the consumption of fuel, the resonance frequency further increases, reducing the possibility of resonance. When the baffle aperture is 118mm, the first-order natural frequency is 36.576Hz, although it remains within the range of the resonance frequency, it is close to the maximum value, and the resonance frequencies above the second order are all higher than 39 Hz, which is completely beyond the range of resonance frequency. Furthermore, as the vehicle travels, the oil level gradually decreases, so the first-order natural frequency can exceed the resonance frequency range. This helps the truck avoid the danger caused by the damage to the diesel tank due to resonance.

For the aperture 38 mm and 78 mm, except for the third-order mode, where the maximum amplitude is on the tank body, the maximum amplitudes for other modes are mainly at the baffle plates. For the aperture 118 mm, the first-order maximum amplitude is at the fuel leakage boss, the fourth-order maximum amplitude is on the tank body, and the maximum amplitudes of other order are mainly at the baffle plates.

The excessive vibration of the baffle and the quality of the welding are the main reasons for failure and fracture. Simply from the perspective of vibration characteristics, the safety of the tank with a baffle aperture 118 mm is higher than the other two apertures, followed by the tank with a baffle aperture 78 mm, and the tank with a baffle aperture 38 mm has the lowest safety. However, it is essential to consider the strength requirements brought about by acceleration and deceleration sloshing, further analysis of the stress distribution and fatigue strength characteristics under the fluid-structure interaction state is needed.

## 7 Conclusion

In this paper, the influences of the structural parameters on the vibration mode were analyzed.

1. The wet modal analysis of the diesel tank was carried out. The comparison between the hammering mode test and simulation modal analysis proved that the maximum error of the data of the structural modal parameters measured by the simulation and the test is 4.8%, which verifies the accuracy of the simulation. It is determined that 22.5 Hz-37.5 Hz is the dangerous resonance frequency range.
2. During the actual trucking process, the oil fill ratio in the tank is basically maintained between 0.1 and 0.9. With decreasing fuel ratio, the natural frequency of the diesel tank decreases, so the natural frequency with fuel ratios of 0.1 to 0.5 is not within the resonance range, but the first-order and second-order modes of the diesel tank with a fuel ratio of 0.9 are 31.131 and 33.326 Hz, respectively, which are within the range of dangerous resonance frequencies.
3. Natural frequency changes along with the different diesel tank structures. With increasing baffle plate number, natural frequency increases, but the change of natural frequency is not obvious when the number of baffles continues to increase. The change of the baffle spacing does not significantly change the natural frequency; the size of the baffle aperture has a significant effect on the natural frequency. Increasing the baffle aperture will effectively increase the natural frequency of the diesel tank. For the diesel tank with a baffle aperture 118 mm, its first-order natural frequency is 36.576 Hz within the range of resonance frequency, close to the maximum value, the second-order and above natural frequencies are all far away from the resonance range, and the first-order natural frequency will appear outside the range with the decrease of the oil, when the truck is driving on the road, the danger caused by the resonance can be avoided.
